# Supplementary material for: The Management of Peutz–Jeghers Syndrome: European Hereditary Tumour Group (EHTG) Guideline
Source: J Clin Med. 2021 Jan 27;10(3):473. doi: 10.3390/jcm10030473 (PMC7865862; doi:10.3390/jcm10030473)
Supplement: Supplementary file 1 [file jcm-10-00473-s001.pdf]

Supplementary Materials:

Key question on cancer risks in Peutz-Jeghers syndrome

What are the cancer risks in PJS?

Literature search:

Embase.com

('Peutz Jeghers syndrome'/de OR (Peutz-Jegher* OR PJS):ab,ti,kw) AND ('neoplasm'/exp OR 'cancer risk'/de OR (cancer* OR neoplas* OR tumor* OR tumour* OR carcinom* OR adenocarcinom*):ab,ti,kw) AND ('risk'/exp OR 'cancer risk'/de OR (risk*):ab,ti,kw) *NOT ((animal/exp OR animal*:de OR nonhuman/de) NOT ('human'/exp)) NOT ('case report'/de OR [conference abstract]/lim) AND [english]/lim*

Medline Ovid

(Peutz-Jeghers Syndrome/ OR (Peutz-Jegher* OR PJS).ab,ti,kf.) AND (exp Neoplasms/ OR (cancer* OR neoplas* OR tumor* OR tumour* OR carcinom* OR adenocarcinom*).ab,ti,kf.) AND (Risk/ OR (risk*).ab,ti,kf.) *NOT (exp animals/ NOT humans/) NOT (news OR congres* OR abstract* OR book* OR chapter* OR dissertation abstract* OR case report*).pt. AND english.lg.*

Cochrane Central

((Peutz-Jegher* OR PJS):ab,ti,kw) AND ((cancer* OR neoplas* OR tumor* OR tumour* OR carcinom* OR adenocarcinom*):ab,ti,kw) AND ((risk*):ab,ti,kw)

**Database searched**	**via**	**Years of coverage**	**References**
Embase	Embase.com	1971-Present	656
Medline ALL	Ovid	1946-Present	335
Cochrane Central Register of Controlled Trials	Wiley	1992-Present	7
**Total**			**998**
**After de-duplication**			**723**

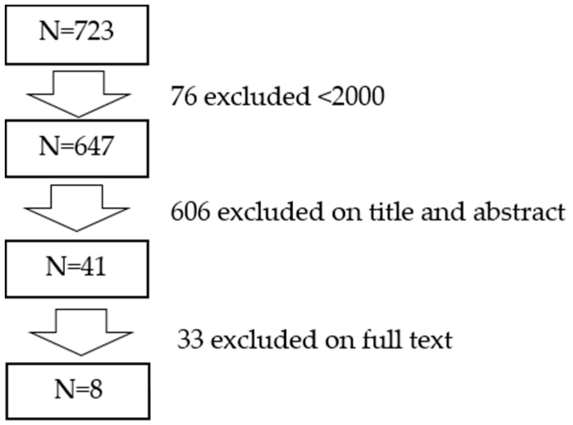

__Key-questions on clinical genetic management__

How often a pathogenic variant in LKB1 is found in patients fulling the clinical PJS criteria?
How often a pathogenic variant in LKB1 is found in patients not fulling the clinical WHO PJS criteria?

Literature search:

Embase.com

('Peutz Jeghers syndrome'/de OR (Peutz-Jegher* OR PJS):ab,ti,kw) AND ('genetic analysis'/exp OR 'genetic heterogeneity'/de OR (((mutation* OR genetic*) NEAR/3 (analys* OR detect* OR heterogeneity))):ab,ti,kw) *NOT ((animal/exp OR animal*:de OR nonhuman/de) NOT ('human'/exp)) NOT ('case report'/de OR [conference abstract]/lim) AND [english]/lim*

Medline

(Peutz-Jeghers Syndrome/ OR (Peutz-Jegher* OR PJS).ab,ti,kf.) AND (Genetic Heterogeneity/ OR (((mutation* OR genetic*) ADJ3 (analys* OR detect* OR heterogeneity))).ab,ti,kf.) *NOT (exp animals/ NOT humans/) NOT (news OR congres* OR abstract* OR book* OR chapter* OR dissertation abstract* OR case report*).pt. AND english.lg.*

Cochrane central

((Peutz-Jegher* OR PJS):ab,ti,kw) AND ((((mutation* OR genetic*) NEAR/3 (analys* OR detect* OR heterogeneity))):ab,ti,kw)

Results:

**Database searched**	**via**	**Years of coverage**	**References**
Embase	Embase.com	1971-Present	235
Medline ALL	Ovid	1946-Present	96
Cochrane Central Register of Controlled Trials	Wiley	1992-Present	1
**Total**			**332**
**After de-duplication**			**244**

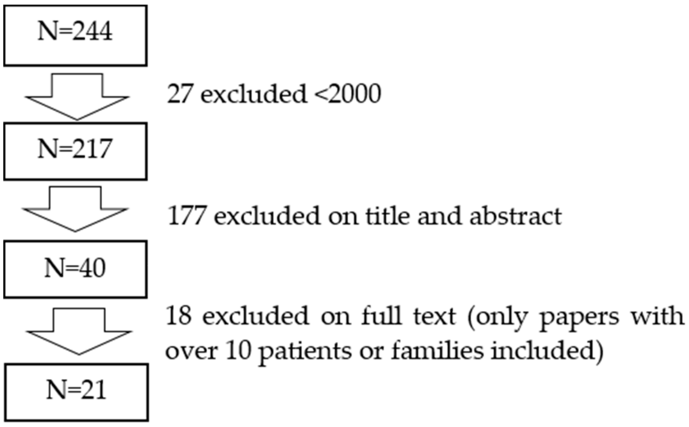

Table S1: Detection rate (DR) of pathogenic variants in PJS patients

**Author (years)**	**Number of tested patients or families /number with pathogenic variants**	**DR %**
Boardman (2000)	6/33	18
Olschwang et al (2001)	24/34	70
Scott et al (2002)	6/14	40*
Lim et al (2003)	17/33	52
Amos et al. (2004)	22/32	69
Aretz et al. (2005)	53/56	94
Chow et al. (2006)	24/33	73
Hearle et al (2006)	25/38	66
Thakur et al (2006)	1/10	10
Volikos et al (2006)	59/76	80
de Leng et al. (2007)	21/23	91
Salloch et al (2010)	16/22	70
Papp et al (2010)	13/13	100
Yang et al (2010)	10/17	64
Borun et al. (2013)	22/41	54
Wang et al (2014)	52/35	67.3
Huang et al. (2015)	11/12	92
Jelsig et al (2016)	18/21	86
Chiang et al (2018)	11/8	73
Jiang et al (2019)	25/34	73.5
Zhao et al (2019)	11/18	61

*The DR could be discussed as some of the variants could be classified as variants of unknown significance

<u>Key-questions on gastrointestinal management</u>

What gastrointestinal surveillance should be offered to persons with PJS?
How should PJS polyps be managed?
What management should be advised in case of one PJS polyp without other signs of PJS , without a family history of PJS and/or without a germline STK11 mutation?
Is there a role for haemoglobin testing in children with PJS under the age of 8 years?

Literature search on key-question:
What management should be advised in case of one PJS polyp without other signs of PJS , without a family history of PJS and/or without a germline STK11 mutation?
Embase.com
('Peutz Jeghers syndrome'/de OR (Peutz-Jegher* OR PJS):ab,ti,kw) AND ((((solitary OR isolated OR single) NEAR/3 (polyp))):ab,ti,kw)

Medline
(Peutz-Jeghers Syndrome/ OR (Peutz-Jegher* OR PJS).ab,ti,kf.) AND ((((solitary OR isolated OR single) ADJ3 (polyp))).ab,ti,kf.)
Cochrane Central
((Peutz-Jegher* OR PJS):ab,ti,kw) AND ((((solitary OR isolated OR single) NEAR/3 (polyp))):ab,ti,kw)

Results:

**Database searched**	**via**	**Years of coverage**	**References**
Embase	Embase.com	1971-Present	53
Medline ALL	Ovid	1946-Present	38
Cochrane Central Register of Controlled Trials	Wiley	1992-Present	0
**Total**			**91**
**After de-duplication**			**60**

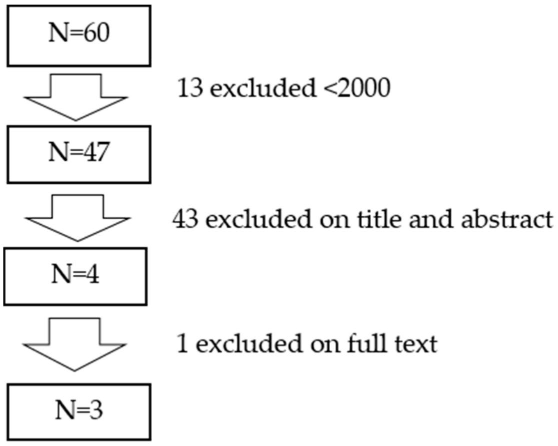

Literature search on key-question:
Is there a role for haemoglobin testing in children with PJS under the age of 8 years?
Embase.com
('Peutz Jeghers syndrome'/de OR (Peutz-Jegher* OR PJS):ab,ti,kw) AND ('anemia'/exp OR 'hemoglobin'/exp OR 'blood analysis'/exp OR (hemoglobin* OR (blood NEAR/3 (test* OR analys*)) OR anemia* OR anaemia*):ab,ti,kw) *NOT ((animal/exp OR animal*:de OR nonhuman/de) NOT ('human'/exp)) NOT ('case report'/de OR [conference abstract]/lim) AND [english]/lim*
Medline
(Peutz-Jeghers Syndrome/ OR (Peutz-Jegher* OR PJS).ab,ti,kf.) AND (exp Anemia/ OR exp Hemoglobins/ OR Blood Chemical Analysis/ OR (hemoglobin* OR (blood ADJ3 (test* OR analys*)) OR anemia* OR anaemia*).ab,ti,kf.) *NOT (exp animals/ NOT humans/) NOT (news OR congres* OR abstract* OR book* OR chapter* OR dissertation abstract* OR case report*).pt. AND english.lg*
Cochrane
((Peutz-Jegher* OR PJS):ab,ti,kw) AND ((hemoglobin* OR (blood NEAR/3 (test* OR analys*)) OR anemia* OR anaemia*):ab,ti,kw)

Results:

Database searched	via	Years of coverage	References
Embase	Embase.com	1971-Present	108
Medline ALL	Ovid	1946-Present	40
Cochrane Central Register of Controlled Trials	Wiley	1992-Present	1
**Total**			**149**
**After de-duplication**			**119**

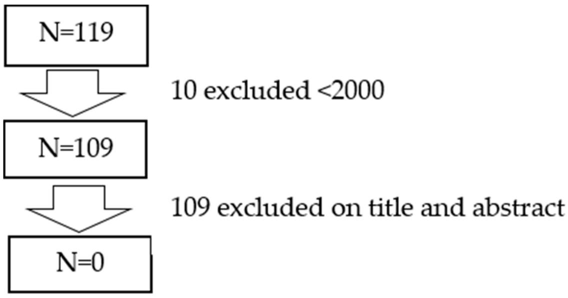

Key-question on surgical management

How should intussusception and intestinal obstruction be managed in patients with PJS?

Literature search:

Embase.com

('Peutz Jeghers syndrome'/de OR (Peutz-Jegher* OR PJS):ab,ti,kw) AND ('intussusception'/exp OR 'emergency surgery'/de OR 'prophylactic surgical procedure'/de OR (intussusception* OR invagination* OR ((acute* OR emergenc* OR prophyla*) NEAR/3 surg*)):ab,ti,kw) *NOT ((animal/exp OR animal*:de OR nonhuman/de) NOT ('human'/exp)) NOT ('case report'/de OR [conference abstract]/lim) AND [english]/lim*

Medline Ovid

(Peutz-Jeghers Syndrome/ OR (Peutz-Jegher* OR PJS).ab,ti,kf.) AND (Intussusception/ OR Prophylactic Surgical Procedures/ OR (intussusception* OR invagination* OR ((acute* OR emergenc* OR prophyla*) ADJ3 surg*)).ab,ti,kf.) *NOT (exp animals/ NOT humans/) NOT (news OR congres* OR abstract* OR book* OR chapter* OR dissertation abstract* OR case report*).pt. AND english.lg.*

Cochrane Central

((Peutz-Jegher* OR PJS):ab,ti,kw) AND ((intussusception* OR invagination* OR ((acute* OR emergenc* OR prophyla*) NEAR/3 surg*)):ab,ti,kw)

Results:

Database searched	via	Years of coverage	References
Embase	Embase.com	1971-Present	147
Medline ALL	Ovid	1946-Present	109
Cochrane Central Register of Controlled Trials	Wiley	1992-Present	3
**Total**			**259**
**After de-duplication**			**177**

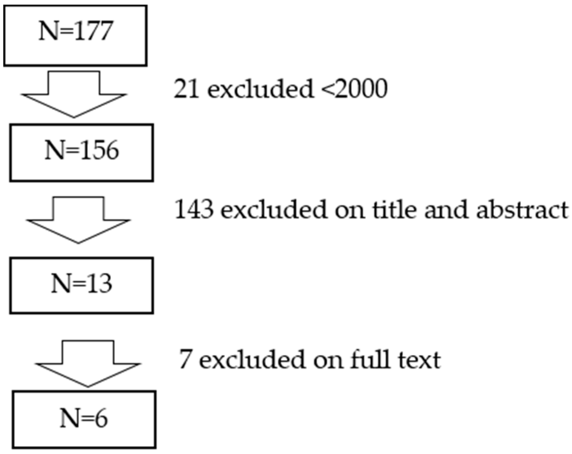

<u>Key-questions on pancreatic management</u>

Should pancreatic surveillance be offered to persons with PJS?
What pancreatic surveillance should be offered?
Does the presence of pancreatic cysts influence the protocol?
Which diagnostic findings trigger a (partial) pancreatectomy?
Is there a place for prophylactic (duodenum-sparing) pancreatectomy in PJS?

Literature search:

Embase.com

('Peutz Jeghers syndrome'/de OR 'high risk patient'/de OR 'high risk population'/de OR (Peutz-Jegher* OR PJS OR ((high-risk) NEAR/3 (patient* OR population* OR individual*))):ab,ti,kw) AND ('pancreas cancer'/de OR 'pancreas carcinoma'/exp OR (((pancrea*) NEAR/3 (cancer* OR carcino* OR adenocarcinom* OR neoplas* OR tumor* OR tumour*))):ab,ti,kw) AND ('screening'/de OR (surveillance OR screening):ab,ti,kw) *NOT ((animal/exp OR animal*:de OR nonhuman/de) NOT ('human'/exp)) NOT ('case report'/de OR [conference abstract]/lim) AND [english]/lim*

Pubmed.gov

(Peutz-Jeghers Syndrome/ OR (Peutz-Jegher* OR PJS OR ((high-risk) AND (patient* OR population* OR individual*)))) AND (Carcinoma, Pancreatic Ductal/ OR Pancreatic Intraductal Neoplasms/ OR Pancreatic Neoplasms/ OR ((pancrea*) AND (cancer* OR carcino* OR adenocarcinom* OR neoplas* OR tumor* OR tumour*)))) AND (Early Detection of Cancer/ OR (surveillance OR screening)) Filters: Clinical Study, Clinical Trial, Guideline, Meta-Analysis, Multicenter Study, Observational Study, Practice Guideline, Randomized Controlled Trial, Review, Systematic Reviews, Humans, English

Cochrane Central
((Peutz-Jegher* OR PJS OR ((high-risk) NEAR/3 (patient* OR population* OR individual*))):ab,ti,kw) AND ((((pancrea*) NEAR/3 (cancer* OR carcino* OR adenocarcinom* OR neoplas* OR tumor* OR tumour*))):ab,ti,kw) AND ((surveillance OR screening):ab,ti,kw)

Results:

**Database searched**	**via**	**Years of coverage**	**References**
Embase	Embase.com	1971-Present	472
Pubmed.gov	Pubmed.gov	1946-Present	429
Cochrane Central Register of Controlled Trials	Wiley	1992-Present	17
**Total**			**918**
**After de-duplication**			**770**

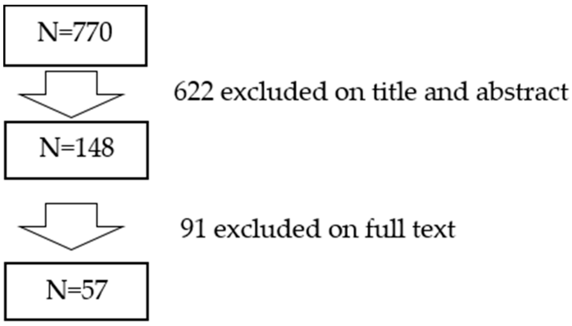

<u>Key-questions on breast management</u>

What breast surveillance should be offered to female patients with PJS?
Is there a role for profylactic mastectomy in females with PJS?

Literature search:
Embase.com
('Peutz Jeghers syndrome'/de OR (Peutz-Jegher* OR PJS):ab,ti,kw) AND ('breast tumor'/exp OR (breast* OR mamma*):ab,ti,kw) *NOT ((animal/exp OR animal*:de OR nonhuman/de) NOT ('human'/exp)) NOT ('case report'/de OR [conference abstract]/lim) AND [english]/lim*
Medline Ovid
(Peutz-Jeghers Syndrome/ OR (Peutz-Jegher* OR PJS).ab,ti,kf.) AND (exp Breast Neoplasms/ OR (breast* OR mamma*).ab,ti,kf.) *NOT (exp animals/ NOT humans/) NOT (news OR congres* OR abstract* OR book* OR chapter* OR dissertation abstract* OR case report*).pt. AND english.lg.*
Cochrane Central
((Peutz-Jegher* OR PJS):ab,ti,kw) AND ((breast* OR mamma*):ab,ti,kw)

Results:

**Database searched**	**via**	**Years of coverage**	**References**
Embase	Embase.com	1971-Present	269
Medline ALL	Ovid	1946-Present	162
Cochrane Central Register of Controlled Trials	Wiley	1992-Present	3
**Total**			**434**
**After de-duplication**			**302**

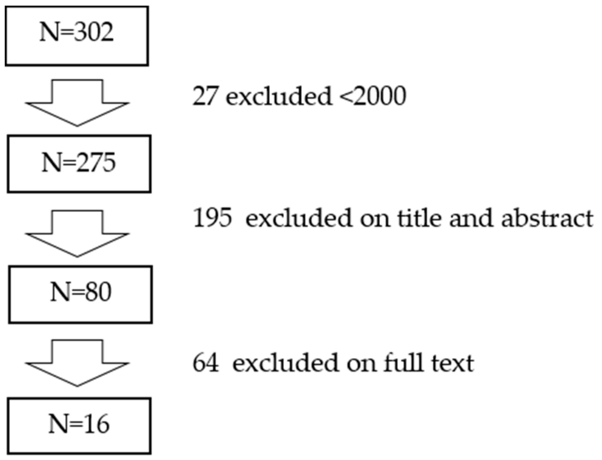

Table S2. Overview of studies on breast cancer in women with (suspected) Peutz-Jeghers syndrome.

Author (year)	Nationality	Type of study	Patients	Breast cancer	Relative/cumulative risk
Choi et al. (2000)	Korea	Retrospective cohort study	16 women with clinical PJS, mean age 23.5 years	1/16 at 35 yrs	
Giardiello et al. (2000)		Meta-analysis	104 women with PJS, 2507.5 person years	11/104 3 bilateral mean 37 yrs (+- SD 11 yrs) range 19-48 yrs	RR 15.2 [95% CI 7.6-27] CR 54% at 64 yrs
Lim et al. (2003)	UK	Retrospective cohort study	19 women with clinical PJS (12 with pathogenic STK11variant)	2/19 at 35 and 52 yrs	CR 29% at age 65 yrs (95% CI: 12–62%) SMR, 13.9 (95% CI: 0.2–50.3, *P*<0.001).
Lim et al. (2004)	Europe, Australia, USA	Retrospective cohort study	131 women with pathogenic STK11 variant	9/131 range 38-62 yrs	RR 7-fold increased CR 8% (95%CI 3-28) at 40 yrs, 11% (95%CI 4-27) at 50 yrs, 32% (95% CI 15-59) at 60 yrs
Hearle et al. (2006)	Europe, Australia, USA	Retrospective cohort study	226 women with clinical PJS (297/419 total patients pathogenic STK11 variant)	16/226 1 male BC range 35-61 yrs	RR: ~6-fold increased CR 8% (95%CI 4-17) at 40 yrs 13% (95%CI 7-24) at 50 yrs 31% (95%CI 18-50) at 60 yrs 45% (95%CI 27-68) at 70 yrs
Mehenni et al. (2006)	Europe, Asia, South-America, North-Africa	Retrospective cohort study	73 women with pathogenic STK11 variant	1/73 range 30-40 yrs	CR 5% by age 40 (0–13)
Beggs et al. (2010)		Systematic review	15 studies	mean 37 yrs range 19–48 yrs	CR 31–54% at age 60 yrs
van Lier et al. (2010)		Systematic review	20 studies	mean 44 yrs	RR 15

					CR 5-8% at age 40 to 32-54% at 60-70 yrs
van Lier et al. (2011)	Netherlands	Cohort study, partly prospective	69 women with clinical PJS (77/133 total patients STK11 pathogenic variant)	6/69 range 46-61 yrs	
Resta et al. (2013)	Italy	Retrospective cohort study	61 women with PJS (99/119 total patients pathogenic STK11 variant)	6/61 range 31-64 yrs	RR 12.5 CR 12.7% at age 40 to 24% at age 60 yrs
Tchekmedyian et al. (2013)	Uruguay	Retrospective cohort study	10 women with clinical PJS	4/10 range 36-65 yrs	
Ishida et al. (2016)	Japan	Systematic review	313 women with clinical PJS	9/313 median 45 yrs range 11-58	Lifetime risk 19.3%
Chen et al. (2017)	China	Retrospective cohort study	155 women with clinical PJS	2/155 >30 yrs	RR 28 (CI 7-113)
Chiang et al. (2018)	Taiwan	Retrospective cohort study	8 women with clinical PJS	2/8 at 48 and 52 yrs	
Fostira et al. (2018)	Greece	Retrospective cohort study	10 women with pathogenic STK11 variant	3/10 2 bilateral mean 33.3 yrs range 31-37 yrs	
Lipsa et al. (2019)	India	Retrospective cohort study	7 women with STK11 variant, 8 women with suspected PJS	4/7 patients with STK11 variant 1 bilateral range 26-60 yrs 5/8 suspected PJS patients* 1 bilateral range 28-54 yrs	

*BRCA1 variant in one patient with breast cancer aged 31 yrs.

Key-question on gynecological management

Should gynecological surveillance be offered to female patients with PJS?
What gynecological surveillance should be offered?
Is PJS an indication for PND?
Is PJS an indication for PGD?

Literature search on key-questions:
Should gynecological surveillance be offered to female patients with PJS?
What gynecological surveillance should be offered?
Embase.com
('Peutz Jeghers syndrome'/de OR (Peutz-Jegher* OR PJS):ab,ti,kw) AND ('neoplasm'/exp OR (cancer* OR neoplas* OR tumor* OR tumour* OR carcinom* OR adenocarcinom*):ab,ti,kw) AND ('gynecological examination'/exp OR 'uterus'/exp OR 'ovary'/exp OR (((gynecolog* OR gynaecolog*) NEAR/3 (surveillance* OR examination* OR screen*)) OR uterus* OR ovar* OR endometri*):ab,ti,kw) *NOT ((animal/exp OR animal*:de OR nonhuman/de) NOT ('human'/exp)) NOT ('case report'/de OR [conference abstract]/lim) AND [english]/lim*
Medline Ovid
(Peutz-Jeghers Syndrome/ OR (Peutz-Jegher* OR PJS).ab,ti,kf.) AND (exp Neoplasms/ OR (cancer* OR neoplas* OR tumor* OR tumour* OR carcinom* OR adenocarcinom*).ab,ti,kf.) AND (Gynecological Examination/ OR exp Uterus/ OR exp Ovary/ OR (((gynecolog* OR gynaecolog*) ADJ3 (surveillance* OR examination* OR screen*)) OR uterus* OR ovar* OR endometri*).ab,ti,kf.) *NOT (exp animals/ NOT humans/) NOT (news OR congres* OR abstract* OR book* OR chapter* OR dissertation abstract* OR case report*).pt. AND english.lg.*
Cochrane Central
((Peutz-Jegher* OR PJS):ab,ti,kw) AND ((cancer* OR neoplas* OR tumor* OR tumour* OR carcinom* OR adenocarcinom*):ab,ti,kw) AND ((((gynecolog* OR gynaecolog*) NEAR/3 (surveillance* OR examination* OR screen*)) OR uterus* OR ovar* OR endometri*):ab,ti,kw)

Results:

**Database searched**	**via**	**Years of coverage**	**References**
Embase	Embase.com	1971-Present	167
Medline ALL	Ovid	1946-Present	119
Cochrane Central Register of Controlled Trials	Wiley	1992-Present	3
**Total**			**289**
**After de-duplication**			**188**

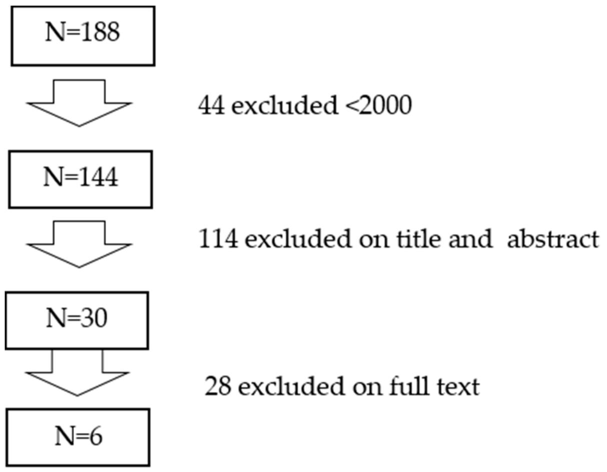

Literature search on key-questions:
Is PJS an indication for PND?
Is PJS an indication for PGD?

Embase.com

('Peutz Jeghers syndrome'/de OR (Peutz-Jegher* OR PJS):ab,ti,kw) AND ('prenatal diagnosis'/exp OR (PGD OR ((prenatal* OR preimplantation* OR pre-implantation*) NEAR/3 (diagnos*))):ab,ti,kw) *AND [english]/lim*

Medline Ovid

(Peutz-Jeghers Syndrome/ OR (Peutz-Jegher* OR PJS).ab,ti,kf.) AND (exp Prenatal Diagnosis/ OR (PGD OR ((prenatal* OR preimplantation* OR pre-implantation*) ADJ3 (diagnos*))).ab,ti,kf.) *AND english.lg.*

Cochrane Central

((Peutz-Jegher* OR PJS):ab,ti,kw) AND ((PGD OR ((prenatal* OR preimplantation* OR pre-implantation*) NEAR/3 (diagnos*))):ab,ti,kw)

Results:

**Database searched**	**via**	**Years of coverage**	**References**
Embase	Embase.com	1971-Present	13
Medline ALL	Ovid	1946-Present	4
Cochrane Central Register of Controlled Trials	Wiley	1992-Present	1
**Total**			**18**
**After de-duplication**			**14**

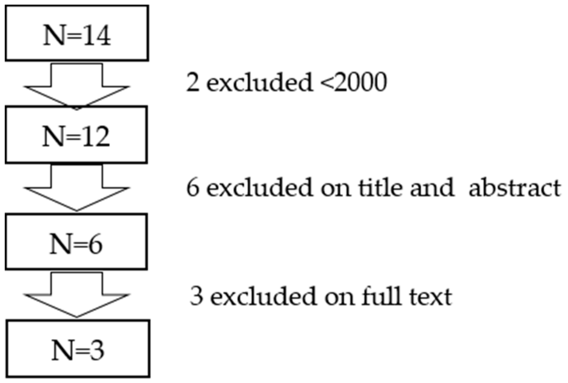
N=14
2 excluded <2000
N=12
6 excluded on title and abstract
N=6
3 excluded on full text
N=3
